# Anti‐inflammatory, antiapoptotic, and antioxidant activity of fluoxetine

**DOI:** 10.1002/prp2.231

**Published:** 2016-04-07

**Authors:** Vitor Caiaffo, Belisa D. R. Oliveira, Fabrício B. de Sá, Joaquim Evêncio Neto

**Affiliations:** ^1^ Department of Animal Morphology and PhysiologyFederal Rural University of PernambucoBrazil; ^2^ Department of PhysiotherapyCaruaruense Association of Higher EducationBrazil

**Keywords:** Antiapoptotic, anti‐inflammatory, antioxidant, Fluoxetine

## Abstract

Fluoxetine is a selective serotonin uptake inhibitor that has been widely used to determine the neurotransmission of serotonin in the central nervous system. This substance has emerged as the drug of choice for the treatment of depression due to is safer profile, fewer side effects, and greater tolerability. Studies have found the following important functions of fluoxetine related to the central nervous system: neuroprotection; anti‐inflammatory properties similar to standard drugs for the treatment of inflammatory conditions; antioxidant properties, contributing to its therapeutic action and an important intracellular mechanism underlying the protective pharmacological effects seen in clinical practice in the treatment of different stress‐related adverse health conditions; and antiapoptotic properties, with greater neuron survival and a reduction in apoptosis mediators as well as oxidative substances, such as superoxide dismutase and hydrogen peroxide. The aim of this study was to perform a review of the literature on the important role of fluoxetine in anti‐inflammatory, cell survival, and neuron trophicity mechanisms (antiapoptotic properties) as well as its role regarding enzymes of the antioxidant defense system.

**Abbreviations**
BDNF: brain‐derived neurotrophic factor
CAT: catalase
CREBcAMP: response element binding protein
FDA: Food and Drug Administration
HOCl: hypochlorous acid
IFN: interferon
IL: interleukin
MDA: Malondialdehyde
NO: nitric oxide
ROS: reactive oxygen species
SOD: superoxide dismutase
TAS: antioxidant status
TNF: tumor necrosis factor

## Introduction

Fluoxetine first emerged in the scientific literature as Lilly 110140 (in hydrochloride form) as a selective serotonin uptake inhibitor (Wong et al. 1974). Following more than 20 years of extensive investigations, serotonin uptake inhibition continues to be the main mechanism of action for fluoxetine, which is a pharmacological agent that has been widely used to determine the neurotransmission of serotonin in the central nervous system (Wong et al. 1995). Evidence from the early 1970s demonstrated that fluoxetine had potential in the treatment of depression (Wong et al. 1974). Since its approval from the US Food and Drug Administration (FDA) on December 29th, 1987, Prozac (the commercial name for fluoxetine) has become the most widely prescribed antidepressant in the world. In 1994, the FDA approved a second clinical use for fluoxetine in the treatment of obsessive‐compulsive disorder and the members of the FDA Advisory Committee unanimously recommended the approval of fluoxetine for the treatment of bulimia in April of that same year (Wong et al. 1995).

Fluoxetine is the drug of choice for the treatment of depression due to its safer profile, fewer side effects, and greater tolerability (Wilde and Benfield 1998). Studies have found important functions of fluoxetine related to the central nervous system. Zhang et al. (2012) discovered a neuroprotective function of this drug against microglial activation due to neurotoxicity in neurons. Novio et al. (2011) demonstrated a positive effect of fluoxetine against stress‐induced oxidative cell damage. Zafir and Banu (2007) also demonstrated the antioxidant potential of this drug stating that such potential could contribute to its therapeutic action and constitutes an important intracellular mechanism underlying the protective pharmacological effects seen clinically in different stress‐related adverse health conditions. Using rats submitted to carrageenan, Abdel‐Salam et al. (2004) also demonstrated the anti‐inflammatory action of fluoxetine and found a response similar to that of standard drugs used to treat inflammatory processes. The administration of fluoxetine has also contributed to a reduction in cell apoptosis. Kolla et al. (2005) demonstrated greater neuron survival and a reduction in oxidative substances, such as superoxide dismutase (SOD) and hydrogen peroxide (H_2_O_2_). Studying children with autism, Makkonen et al. (2011) demonstrated in increase in the concentration of insulin‐like growth factor (IGF‐1), which is an important neurogenic factor of the central nervous system, following the use of fluoxetine. The authors also state that the increase in IGF‐1 must play an important role in brain development and the modulation of neuronal processes.

The aim of this study was to perform a review of the literature on the important role of fluoxetine in anti‐inflammatory, cell survival, and neuron trophicity mechanisms (antiapoptotic properties) as well as its role regarding enzymes of the antioxidant defense system.

### Anti‐inflammatory action

It has recently been demonstrated that depression is associated with the activation of the inflammatory response system (Makkonen et al. 2011), as evidence has demonstrated an increase in the production of proinflammatory cytokines, such as interleukin‐1*β* (IL‐1*β*), IL‐6 and interferon‐*γ* (IFN‐*γ*) (Maes 1999; Makkonen et al. 2011). An increase in the production of IL‐1 and IL‐2 has been found in the supernatant of mitogen‐stimulated splenocyte cultures from rats submitted to chronic stress and rat depression models (Seidel et al. 1995; Kubera et al. 1996). Yirmiya (1996) and Maes (1999) suggest that the increase in the production of proinflammatory cytokines may play a role in the etiology of depression. Indeed, IL‐1, IL‐6, and IFN‐*γ* in experiments involving animals and humans have been found to produce behavioral changes and symptoms similar to those found in depression, such as an inability to feel pleasure, anorexia, weight loss, withdrawal from social situations, psychomotor retardation, a lack of energy, irritability, and sleep disorders (Makkonen et al. 2011).

As proinflammatory cytokines are involved in the etiology of depression, one may expect that antidepressants have negative immunomodulating effects. Prolonged treatment with antidepressants suppresses the response in the acute phase among patients with depression (Yirmiya 1996) as well as rat models of depression involving mild chronic stress (Maes et al. 1997). In experimental inflammation models, fluoxetine has been found to exert anti‐inflammatory and pain relief effects. However, the mechanism by which this drug reduces inflammation remains unclear (Song and Leonard 1994).

Abdel‐Salam et al. (2004) induced edema in rat paws with the administration of carrageenan and used fluoxetine (either alone or in combination with non‐steroidal anti‐inflammatory drugs, other antidepressants or melatonin) and found that the intraperitoneal administration fluoxetine alone suppressed the edema in a dose‐dependent fashion). Likewise, when fluoxetine was administered in conjunction with non‐steroidal anti‐inflammatory drugs (indomethacin, celecoxib, and rofecoxib), other antidepressants (imipramine and trazodone) or melatonin, the combination led to a reduction in inflammation, suggesting that fluoxetine is a useful drug for combating inflammatory processes, especially when administrated in conjunction with other specific drugs.

The administration of desipramine in rats increases the capacity of splenocytes to produce the negative immune‐regulating cytokine IL‐10 (Bianchi et al. 1994). Ex vivo studies have demonstrated that antidepressants inhibit the secretion of IL‐1*β*, IL‐2, tumor necrosis factor alpha (TNF‐*α*), and IFN‐*γ* as well as inhibit the proliferative activity of T cells and the cytotoxic activity of natural killer cells (Bianchi et al. 1994; Makkonen et al. 2011). The coincubation of whole blood from health human subjects and either clomipramine, which is a tricylcic antidepressant, or sertraline, which is a selective serotonin uptake inhibitor, diminishes the production of IFN‐*γ* and increases the production of IL‐10 (Makkonen et al. 2011). IFN‐*γ* is a proinflammatory cytokine produced by activated T lymphocytes and natural killer cells that stimulates the production of IL‐1 and IL‐6 (Xia et al. 1996). IL‐10 is produced by T‐helper lymphocytes, B lymphocytes, and monocytes and has important anti‐inflammatory and immunosuppressive properties through the suppression of IFN‐*γ* and other proinflammatory cytokines (Xia et al. 1996). Thus, the IFN‐*γ* to IL‐10 ratio produced by immune cells is of fundamental importance to the determination of the capacity to activate or inhibit the functions of monocytes and T lymphocytes (Cavaillon 1996).

Analyzing young adults and elderly individuals with treatment‐resistant depression, Kubera et al. (2001) found that the administration of fluoxetine significantly reduced the production of IFN‐*γ*, especially in the elderly subjects, and that the production of IL‐10 was significantly greater among the individuals treated with this antidepressant.

In microglia subjected to activation by lipopolysaccharide, Liu et al. (2011) demonstrated that fluoxetine promotes a decrease in production of IL‐6, TNF‐*α*, and nitric oxide (NO). These authors also demonstrated that the molecular mechanism that fluoxetine acts is through a decrease in the gene expression observed by reducing the transcription levels of mRNA of IL‐6 and TNF‐*α*. Furthermore, fluoxetine may also act by inhibiting the phosphorylation of mitogen activated protein kinase, important signaling pathway of proinflammatory cytokines, and activation of nuclear factor kappaB (NF‐kB), a significant inflammatory signaling molecule. Likewise, Ghosh et al. (2015) found a change in the levels of IL‐4, IL‐6, IL‐10, and IL‐12 in macrophages maintained in a culture of fluid tumor cells. This change in the levels of these cytokines shows an altered activation of macrophages with a possible phenotypic change in M1 macrophages (classic) to M2 (changed). After supplementation with fluoxetine, these authors demonstrated significant reversal of IL‐4 levels, IL‐6, and IL‐10 for similar to the control group. These authors declare that fluoxetine can act on the reversal of polarization of modified macrophages by inhibition of NF‐kB, strongly suggesting that fluoxetine can be an effective agent able to reprogram macrophages in favor of the host undergoing inflammatory conditions.

Thus, these studies cited demonstrate the importance of fluoxetine to the activation of the anti‐inflammatory response system.

### Antiapoptotic action

The role of intracellular signaling molecules, transcription factors, and chromatin‐modifying enzymes has been highlighted in the physiopathology and treatment of mood disorders (Kubera et al. 2001). Special attention has been given to apoptotic processes, which are programmed and controlled by the balance between proapoptotic (e.g., BAD) and antiapoptotic (e.g., Bcl‐2) molecules (Covington et al. 2010).

Bcl‐2 attenuates apoptosis by promoting cell survival as well as axon growth and neuron regeneration (Cory and Adams 2002). The expression of Bcl‐2 is controlled by different factors involved in survival pathways, such as brain‐derived neurotrophic factor (BDNF) and the response element to AMPc cAMP response element‐binding protein (CREB). This expression can be regulated by stress, antidepressants, and antipsychotics (Park et al. 2006; Drzyzga et al. 2009; Hammonds and Shim 2009). Moreover, it has been demonstrated that antidepressants overregulate the expression of Bcl‐2 (Xu et al. 2003; Kock et al. 2009). Curiously, the stimulation of BDNF also activates the protein kinase B, which is also known as Akt (Chiou et al. 2006). The Akt protein complex is involved in the neuron structure, cell survival, and cell death through apoptosis and also stimulates the expression of Bcl‐2, thereby inhibiting apoptosis (Lawlor and Alessi 2001; Huang and Reichardt 2003).

Using fluoxetine and olanzapine either alone or combined, Réus et al. (2012) found an increase in Akt, CREB, BDNF, Bcl‐2, and BAD in the frontal cortex, hippocampus, and striatum nucleus of adult rats. These areas are very much involved in mood disorders and depression. Bcl‐2 belongs to a family of proteins that regulate programmed cell death. The control of the apoptotic process is achieved by the balance between proapoptotic (e.g., BAD and BAX) and antiapoptotic (e.g., Bcl‐2 and Bcl‐XL) proteins (Hammonds and Shim 2009). An increase in proapoptotic over antiapoptotic proteins is associated with greater susceptibility to cell death (Lawlor and Alessi 2001). Thus, Réus et al. (2012) suggest an antiapoptotic effect of treatment with fluoxetine and olanzapine.

It has been demonstrated that Bcl‐2 is a repressor of neuron death (Myers et al. 1995; Lindsten et al. 2005). Both Bcl‐2 and BAD regulate the release of cytochrome c, which plays a role in the onset of apoptosis (Li et al. 1997). Agostinho et al. (2011b) evaluated the effects of fluoxetine and olanzapine on mitochondrial respiratory chains and found that the two drugs, either alone or in conjunction, alter the activity of these chains in the brain of rats. Moreover, acute treatment with fluoxetine alters the activity of the enzyme citrate synthase and both acute and chronic treatments modify the activity of the enzyme creatine kinase (Agostinho et al. 2009, 2011a). These enzymes are involved in cell metabolism and the relationship between fluoxetine and energy metabolism has been clearly demonstrated, which is correlated with neuropsychiatric disorders (Ben‐Shachar and Karry 2008; Quiroz et al. 2008).

Besides controlling cell survival, the antiapoptotic protein Bcl‐2 also exerts a neurotrophic effect (Jonas et al. 2003). Studies report that some antidepressants (e.g., fluoxetine) and stress regulate neurotrophic factors, such as CREB and BDNF, which are involved in cell survival pathways and control the expression of proapoptotic and antiapoptotic proteins (Manji et al. 2001). Kosten et al. (2008) found that repeated, unpredictable stress reduces the mRNA levels of Bcl‐2 in the central nucleus of the amygdala in the cingulate gyrus and frontal cortices. In contrast, treatment with antidepressants, such as fluoxetine, reboxetine, and tranylcypromine, overregulates the expression of Bcl‐2 in the same areas of the brain (Quiroz et al. 2008). Huang et al. (2007) also found that desipramine inhibits the induction of IL‐1b and IL‐6 mediated by the modulation of Bcl‐2. Moreover, it has been demonstrated that the administration of olanzapine regulates the expression of Bcl‐2 RNAm in the hippocampus of rats (Bai et al. 2004).

Likewise, Ghosh et al. (2015) have demonstrated an increase in apoptosis in T cells as evidenced by increased annexin‐positive cells and cells with mitochondrial depolarization. These changes represent the development of an immunosuppressive condition caused by fluid tumor used. These adverse conditions were reversed by supplementation with fluoxetine, strengthening the immunoregulatory role of this antidepressant. In order to assess the status of pro / antiapoptotic markers within these T cells, this study evaluated the intracellular level of caspase‐3 and Bcl‐2, respectively, and it was found a reduction in caspase‐3 expression with concomitant increase in the level of Bcl‐2 in these cells with supplementation of fluoxetine. This result suggests an antiapoptotic role of fluoxetine.

The overregulation of CREB regulates the transcription of Bcl‐2 (Lonze and Ginty 2002). The expression of Bcl‐2 is also stimulated by the activation of Akt, thereby inhibiting apoptosis (Gerber et al. 1999). Studies have demonstrated that cortical regions exhibit a reduction in the function of Akt among depressed individuals (Hsiung et al. 2003; Karege et al. 2007) and reduced Akt levels are also found in the brain of schizophrenic patients (Emamian et al. 2004). Réus et al. (2012) found that the combination of fluoxetine and olanzapine leads to an increase in Akt levels in the prefrontal cortex. Nakano et al. (2010) found that treatment with BDNF also stimulates the phosphorylation of Akt. Réus et al. (2012) found that high levels of Bcl‐2 due to the administration of fluoxetine may be involved in the activation of CREB through signaling by Akt. Indeed, the Bcl‐2 promoter gene has an element that is responsive to AMPc (CRE), which, when phosphorylated (CREB), binds to mRNA and increases the transcription of the antiapoptotic protein (Belkkhiri et al. 2008).

Overregulated CREB can activate targets, such as BDNF, following the use of antidepressants (Nibuya et al. 1996). Treatment with the combination of fluoxetine and olanzapine has been found to raise BDNF and CREB levels in rats; however, the administration of fluoxetine alone at a dose of 12.5 mg/Kg leads to a greater increase in BDNF (Réus et al. 2012). In contrast, Shishkina et al. (2012) found that levels of BDNF and proapoptotic proteins (e.g., BAX) were unaltered with the administration of fluoxetine or due to stress. However, high BDNF levels were found in the dentate gyrus of the hippocampus when combining treatment with fluoxetine and ketanserin, which is an antagonist of the serotoninergic receptor 5‐HT(2A) (Pilar‐Cuéllar et al. 2012). The authors suggest that the effect of fluoxetine on BDNF is related to the type of treatment, administration protocol, and area of the brain. Réus et al. (2012) also found that the effects of fluoxetine are dependent on the dosage and area of the brain. The authors explain this dependent effect by the fact that the brain has dissimilar areas and distinct areas have different proteins and cell metabolisms.

Although several studies show antiapoptotic effects of fluoxetine, Djordjevic et al. (2011) found a positive relationship of Bax/Bcl‐2 in rats livers stress induced and treated with fluoxetine. These authors report that the prevalence of Bax in relation to the Bcl‐2 can be interpreted as an activation of the apoptotic response under treatment with fluoxetine. These authors also found increased DNA fragmentation in animal cells treated with fluoxetine. This DNA fragmentation can be due to a greater decrease in Bcl‐2 than the increase in Bax. These results corroborate the findings of Zhai et al. (2009) that demonstrated an increased apoptosis after administration of fluoxetine. Due to this apoptotic potential of fluoxetine, Serafeim et al. (2003) considers it as a promising therapeutic agent for Burkitt's lymphoma.

### Antioxidant action

Central nervous tissue has a high percentage of phospholipids, such as arachidonic acid, docosahexaenoic acid, inositol phosphate, and diacylglycerol, which are easily peroxidated, consequently generating reactive oxygen species (ROS). These phospholipids are generated as secondary messengers by neurotransmitters (e.g., dopamine, serotonin, glutamate, and acetylcholine) normally involved in the etiopathology of diseases of the central nervous system (Mahadik and Evans 2003).

Free radicals are very reactive species that have an unpaired electron. This large group of molecules is mainly represented by the superoxide radical (O_2_•), peroxyl radical (ROO•), hydroxyl radical (OH•), and NO•. These molecules and their respective byproducts, such as hydrogen peroxide (H_2_O_2_) and hypochlorous acid (HOCl), are considered ROS (Drõge 2002; Valko et al. 2007). The overproduction of ROS results in an imbalance in prooxidant and antioxidant processes, which lead to the phenomenon known as oxidative stress. Oxidative stress is harmful to lipids, proteins, nucleic acids, and other cell structures. This process alters the normal cell metabolism and can even lead to cell death. The process of lipid peroxidation, which also results in the production of free radicals, is the most well‐known harm caused by oxidative stress (Hwang and Kim 2007). Malondialdehyde (MDA) is the most widely studied product of lipid peroxidation. This aldehyde is a highly toxic molecule that interacts with proteins and DNA and is considered mitogenic. Studies addressing lipid peroxidation in patients with depression describe an increase in MDA levels and other products of lipid peroxidation (Tsuboi et al. 2006; Sarandol et al. 2007; Galecki et al. 2009).

Different biochemical processes can raise the number of ROS, characterizing some central nervous system conditions, such as depression. One of these processes is the increase in glutaminergic transduction and an increase in the concentration of glutamate to toxic levels (Lee et al. 2002). The prolonged activation of neurons by glutamate can be harmful due to the consequent production of ROS (Hendriks et al. 2005).

Studies have found that individuals with depression have high levels of proinflammatory substances, such as IL‐1, IL‐2, IL‐6, and TNF‐*α* (Schiepers et al. 2005; Raison et al. 2006). These cytokines, especially IL‐1, increase the activity of the hypothalamus–hypophysis–adrenal system, thereby increasing the number of free radicals (Lee et al. 2002). A depressive state stimulates immune cells, such as neutrophils, macrophages, monocytes, astrocytes, and microglia (Tsuboi et al. 2006). These cells interact with proinflammatory cytokines, resulting in the production of free radicals (Guzik et al. 2003). Based on the inflammatory components involved in the etiology of depression, researchers have suggested that antidepressants act on proinflammatory cytokines (Brustoilim et al. 2006).

There is little information regarding the influence of fluoxetine on antioxidant enzymes. While some studies suggest that this antidepressant restores the antioxidant capacity in the brain (Bilici et al. 2001; Novio et al. 2011), Djordjevic et al. (2011) suggested that the fluoxetine affect liver antioxidant system of rats and other studies report that such therapy does not significantly alter this capacity in patients with depression (Tsuboi et al. 2006).

The primary antioxidant defense system involves the coordinated effects of some enzymes, such as SOD, catalase (CAT), GPx, and GR, which have consistently been studied in individuals with depression (Ng et al. 2008). Using mice submitted to stress, Moretti et al. (2012) found a reduction in the activity of CAT and GR, especially in the hippocampus, as well as GR in the cerebral cortex, indicating a change in the antioxidant defense system in the nervous system of these animals. A reduction in CAT activity is associated with a large amount of H_2_O_2_ available to react with transcription metals and generate the most harmful radical (hydroxyl), resulting in an increase in lipid peroxidation and subsequent neuron damage (Meister and Anderson 1983). As glutathione is the main non‐protein antioxidant molecule and a major redox regulator that protects the nervous system against ROS (Moretti et al. 2012), reductions in glutathione levels and GR activity lend support to the notion that stress induces an increase in oxidative damage in the brain of rats. Based on such evidence, Moretti et al. (2012) suggest that the significant oxidative damage is found in authors' studies was evidenced by the increase in lipid peroxidation in the hippocampus and cerebral cortex, resulting from reductions in CAT (hippocampus and cerebral cortex) and GR (hippocampus) activity as well as glutathione levels (cerebral cortex). Using ascorbic acid and fluoxetine as treatment, the same authors demonstrated prevention against induced stress, suggesting a reduction in lipid peroxidation in the hippocampus, mainly by avoiding a reduction in CAT activity. These findings are consistent proof of the antioxidant effects of antidepressants, as the damage evaluated in plasma, serum, and blood cells was reversed following treatment with classic antidepressants, such as fluoxetine, suggesting that the antioxidant properties of these medications contribute to an improvement in clinical aspects (Bilici et al. 2001; Khanzode et al. 2003; Herken et al. 2007).

However, Djordjevic et al. (2011) found a decrease in SOD activity, a noninterference in the CAT activity, increased activity of GPx, increased GR activity, and an increase in the antioxidant status (TAS) in the liver of rats subjected to chronic stress and treated with fluoxetine. According to Souza et al. (1994), fluoxetine directly affects the structure of the mitochondrial membrane of hepatocytes, generating an increase lipid peroxidation. With decreased SOD, primarily responsible for the prevention of lipid peroxidation, GPx rises and emerges as a compensatory response to increased peroxidation. The antioxidant effect of fluoxetine reported in many studiescan also be due to the fact that this drug increases serotonin levels which exhibits antioxidant effects (Huether and Schuff‐Werner 1996). However, Djordjevic et al. (2011) claim that the increase in TAS can serve as an indicator of increased liver oxidative damage.

Adverse stimuli, such as stress and anxiety, can induce peripheral oxidative stress by increasing the production of ROS in lymphocytes, granulocytes, and monocytes (Rammal et al. 2008). Analyzing mice treated with fluoxetine, Novío et al. (2011) found a partial improvement in the adverse effects caused by induced stress. This improvement is associated with the restorative capacity of fluoxetine with regard to endogenous components of the antioxidant defense system (SOD, CAT, and diaphorase) as well as the restoration of non‐enzymatic antioxidant components, such as glutathione, thereby demonstrating that fluoxetine is capable of attenuating oxidative damage produced by stress in the peripheral immune system.

The oxidant/antioxidant relationship is an important determinant of cellular immunological function (Rammal et al. 2008). A reduction in antioxidant status favors the accumulation of free radicals. This leads to oxidative stress and the possible apoptosis of immune cells, such as neutrophils (Meydani et al. 1995), which can be prevented by CAT and glutathione. Considering the fundamental role of immune cells in the protection of the organism, these findings lend support to theory that stressed animals are predisposed to recurring infections and chronic inflammation as well as other adverse health conditions (Rahman and Macnee 2000; Kowalska et al. 2003; Halliwell 2006).

Novío et al. (2011) and Djordjevic et al. (2011) found that fluoxetine alone promotes a change in the antioxidant status of non‐stressed animals, meaning that this antidepressant may not alter the antioxidant defense status in the absence of oxidative stress. This finding suggests that fluoxetine has an antioxidant effect only under conditions of oxidative damage.

## Final Considerations

The findings of this study demonstrate that fluoxetine exerts a significant effect on the improvement of defense systems, with the overregulation of the production of anti‐inflammatory cytokine, the production of antioxidant enzymes, and the modulation of the cell apoptosis cascade. This drug also acts on the inhibition of the release of proinflammatory cytokines as well as the reduction in the expression of prooxidant enzymes and free radicals. Thus, this study reports the important anti‐inflammatory, antiapoptotic, and antioxidant functions of fluoxetine and suggests that the clinical benefits of its administration are related to the cell‐modulating functions in the central and peripheral nervous systems.

## Disclosure

The authors declare no conflicts of interest.
